# AngioMT: A MATLAB based 2D image-to-physics tool to predict oxygen transport in vascularized microphysiological systems

**DOI:** 10.1371/journal.pone.0299160

**Published:** 2024-05-15

**Authors:** Tanmay Mathur, James J. Tronolone, Abhishek Jain

**Affiliations:** 1 Department of Biomedical Engineering, Texas A&M University, College Station, Texas, United States of America; 2 Department of Medical Physiology, College of Medicine, Texas A&M Health Science Center, Bryan, Texas, United States of America; 3 Department of Cardiovascular Sciences, Houston Methodist Research Institute, Houston, Texas, United States of America; KIST: Korea Institute of Science and Technology, GERMANY

## Abstract

Microphysiological models (MPS) are increasingly getting recognized as *in vitro* preclinical systems of pathophysiology and drug discovery. However, there is also a growing need to adapt and advance MPS to include the physiological contributions of the capillary vascular dynamics, because they undergo angiogenesis or vasculogenesis to deliver soluble oxygen and nutrients to its organs. Currently, the process of formation of microvessels in MPS is measured arbitrarily, and vascularized MPS do not include oxygen measurements in their analysis. Sensing and measuring tissue oxygen delivery is extremely difficult because it requires access to opaque and deep tissue, and/or requires extensive integration of biosensors that makes such systems impractical to use in the real world. Here, a finite element method-based oxygen transport program, called AngioMT, is built in MATLAB. AngioMT processes the routinely acquired 2D confocal images of microvascular networks *in vitro* and solves physical equations of diffusion-reaction dominated oxygen transport phenomena. This user-friendly *image-to-physics* transition in AngioMT is an enabling tool of MPS analysis because unlike the averaged morphological measures of vessels, it provides information of the spatial transport of oxygen both within the microvessels and the surrounding tissue regions. Further, it solves the more complex higher order reaction mechanisms which also improve the physiological relevance of this tool when compared directly against *in vivo* measurements. Finally, the program is applied in a multicellular vascularized MPS by including the ability to define additional organ/tissue subtypes in complex co-cultured systems. Therefore, AngioMT serves as an analytical tool to enhance the predictive power and performance of MPS that incorporate microcirculation.

## Introduction

Since the passage of the FDA Modernization Act 2.0, that removes the mandatory requirements of animal studies in drug discovery, microphysiological systems (MPS) have been proposed as a valuable *in vitro* discovery platform [1,2]. MPS models like vascular organ-chips or vessel-chips have served as blood vessel surrogates to study a range of large vessel disorders like atherosclerosis, venous thrombosis, sickle cell disease etc. However, there is also a growing need to adapt and advance MPS to include the physiological contributions of capillary or microvascular dynamics, because they account for most of the vascular transport of soluble oxygen and nutrients to organs and deep tissues. Self-assembly of nascent endothelial cells through *angiogenesis* or *vasculogenesis* is hence increasingly being employed in MPS models to vascularize tissue-chips to enable a physiologically-relevant organ-level platform.

The microvascular niche functions as the mediator of transport of soluble factors and nutrients from the cardiovascular system to deep tissues [3,4]. Efficient removal of reaction biproducts and toxins plays an important role in maintaining homeostasis or when they are altered in various diseases including cancer, type 1 diabetes, and physical injuries like hemorrhage [5,6]. The ability of microvascular networks to effectively penetrate regions of deep lying tissues helps the body maintain oxygen saturation and prevents instances of hypoxia [7]. Therefore, evaluation of the performance of the microvasculature is critical in vascular biology as impaired transport in microvessels is the primary cause of organ failures and necrosis [8,9]. Specifically in the context of engineered vascular systems like organ-chips and vascularized organoids, the ability to measure comparative performances of different vascular conditions is essential [10,11]. Measuring the transport of dissolved nutrients, growth factors, cytokines, drugs etc. in a spatiotemporal manner can serve as a better metric to evaluate microvascular performance in contrast to arbitrary vessel metrics like vessel length, area, branchpoints etc. However, concentration measurements in real-time are limited, cumbersome and extremely difficult because it requires access to opaque and deep tissue, and/or requires extensive integration of biosensors making such methods impractical to use in the real world. Since reconstruction of the microvasculature in vascularized MPS is relatively easier and requires standard immunofluorescence staining followed by confocal imaging, approaches that may (i) leverage the high throughput imaging data generated from multiplexed *in vitro* experiments and (ii) recapitulate the exact vessel architecture to predict unique oxygen distributions between networks are highly desirable.

In this study, we present AngioMT (Angio Mass Transfer), a MATLAB-based computational solver, for investigating vascular and surrounding tissue oxygen transport phenomena from routinely acquired 2D confocal images. This platform includes complex microvascular geometries, multi-tissue microenvironment and high-order reaction kinetics that may be physiologically-relevant, but cannot be easily accommodated in existing commercial or open-source computational transport packages. This image-to-physics software is expected to serve as a digital non-invasive sensor of a native or model of a vascularized organ under conditions of health, disease or surgical treatments.

## Materials and methods

### Data acquisition

The imaging dataset used in the study was obtained in house and have been previously published [12]. Briefly, devices with microvascular networks were fixed with 4% paraformaldehyde (AAJ19943K2, Fisher Scientific) for 15 minutes at room temperature, washed, and permeabilized with 0.1 vol% TritonX- 100 (T8787-100ML, Sigma Aldrich) in 3% bovine serum albumin (BSA) (BP9706100, Fisher Scientific) for 15 minutes at room temperature. Devices were washed, then stained using AlexaFluor-488-conjugated CD-31 antibody (303110, Biolegend; San Diego, CA 92121, USA) diluted 1:100 in 3% BSA overnight at 4°C. Devices were washed and stained with rhodamine phalloidin (R415, Thermo Fisher) for 1 hour at room temperature, washed, and counterstained with Hoescht (H2570, Thermo Fisher) for 10 minutes at room temperature. Vascularization region of each sample was imaged using Z-stack and tiling acquisition with a Zeiss Axio Observer Z1 Inverted Microscope (Zeiss; Thornwood, New York 10594, USA). Stitched images were orthogonally projected in the XY direction and cropped to and area of 1x1 mm. A total of 500 images were acquired spanning various experimental conditions including growth factor, matrix stiffness, cell density variations. The images were exported as 16-bit TIFF files respectively.

### Image processing

16-bit grayscale images were acquired from fluorescence microscopy and were then binarized using a threshold value of 0.2 for all images. Binarization resulted in vascular networks displayed as white pixels (intensity = 1) and hydrogel regions being displayed as black pixels (intensity = 0).

### Image segmentation

AngioMT has been designed to accommodate multiple tissue types within the same ROI. Hence segmentation of binarized images is required to identify and differentiate distinct zones (disconnected vessels, connected vessels, surrounding matrix, other organ tissues etc.) present. AngioMT calculated and assigned unique grayscale values to these “N” distinct domains in increments of 1/_(*N*-1)_. For example, for images with only connected vessels, disconnected vessels and surrounding tissue region (N = 3), AngioMT assigned 3 grayscale values in the range [0, 0.5, 1]. Similarly, for images with additional tissue regions (N = 4) within the ROI, grayscale values assigned were in the range [0, 0.33, 0.66, 1].

### Multi-domain mesh generation

The segmented and recolored images were then meshed using an open-source Delaunay Triangulation scheme called *Im2mesh* developed by Jiexian Ma et al [13]. This script reads greyscaled images with different grayscale values and meshes the image into distinct domains based on the number of greyscale value. Since our images had three distinct grayscale values; 1 for connected vessels, 0.5 for disconnected vessels and 0 for surrounding tissue regions (fibrin hydrogel in our case), the obtained mesh had three distinct domains.

### Model parameters

Table 1 Shows all the values of all parameters used in the study.

**Table 1:** List of parameters and corresponding values used in the study.

Parameter	Value	Ref.
$D_{O_{2}}^{vessel}$	3 x 10^−9^ m^2^/s	[14]
$D_{O_{2}}^{tisue}$	2 x 10^−9^ m^2^/s	[15,16]
$P_{O_{2}}^{vessel}$	2.75 x 10^−14^ mol/(m^2^s)	[17]
*R*_*max*_	0.06 x 10^−12^ mol/(m^3^s)	[14]
*K*_*M*_	1 x 10^−3^ mol/m^3^	[14,16]

## Results & discussion

### Design and physical formulation of the AngioMT software

**Fig 1:**
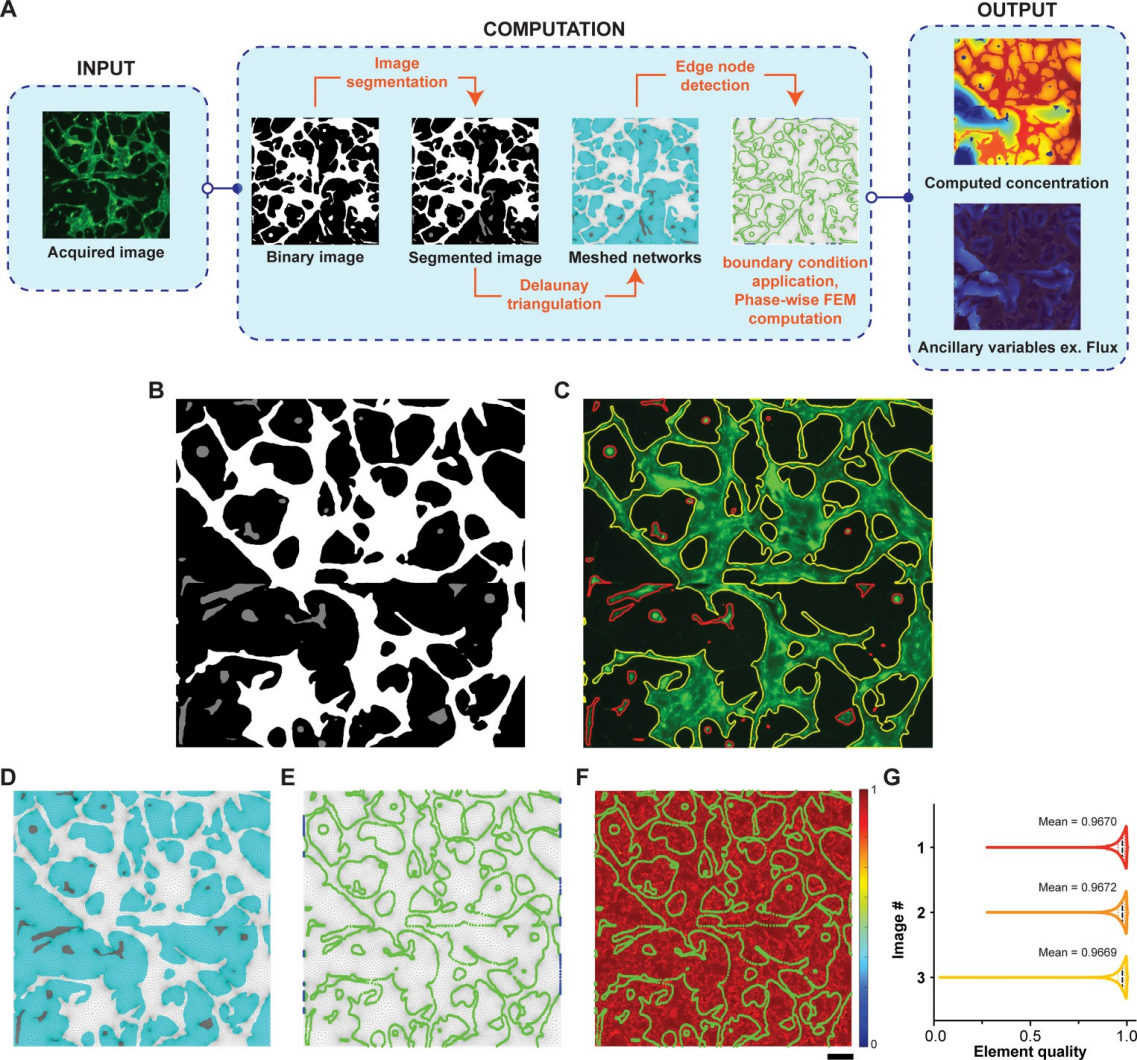
AngioMT process flow, mesh quality assessment and computational validation. **(A)** Images are acquired using fluorescence microscopy and are then binarized. The binarized images are segmented using image processing principles to divide the region into distinct domains. The segmented images are then meshed using Delaunay triangulation and edge nodes are identified. Boundary conditions are applied to the appropriate domains and oxygen concentration is computed for all nodes. Once concentration values are obtained, ancillary variable like flux can be calculated. **(B)** Resulting segmented image with disconnected ‘islands’ shaded as gray. **(C)** Overlay of the boundaries of segmented regions over the original fluorescence image. Connected and inlet domains were labelled with yellow boundaries while disconnected islands were labeled with a red boundary. Representative images describing the **(D)** meshed regions being divided into connected vessel (white), disconnected vessels (gray) and tissue (cyan) domains; **(E)** the edge nodes between tissue and vessel domains (green), as well as the inlet nodes (blue) for applying boundary conditions; **(F)** element quality map for the entire meshed region overlayed with the edge nodes (green) (scale bar: 100 μm). **(G)** Element quality analysis of computational mesh created on variable representative images of vascular networks.

Since 2D fluorescence imaging of vascularized networks are available in literature [18], or they can be acquired by standard microscopy in most labs, we were inspired to start our analysis using binarized 2D confocal images of microvascular networks **(Fig 1A)**. Once we extracted the data from the images in AngioMT, each distinct region in the binarized image was labeled using the inbuilt ‘bwlabel’ feature in MATLAB. Labelled images were then processed and labeled regions were then segmented into three distinct domains: connected vessels (vessels connected to at least one inlet face), solid tissue with no vessels, and disconnected vessels. We independently meshed these regions in AngioMT which was later used to define transport and kinetic properties in the AngioMT routine, and a new greyscale value was assigned to the disconnected vessel domain (intensity = 0.5, **Fig 1B**). Overlaying the segmented domains over the original fluorescence image shows accurate detection of disconnected vessel domains **(Fig 1C)**. Regions outside the detected domains are treated as tissue domains owing to the binarization threshold used. These segmented images were then meshed using the *Im2mesh* routine [13] which performs Delaunay Triangulation over the entire image based on the different grayscale values defined in the image, resulting in a composite mesh that was later utilized for generating finite element formulations of the mass transport dynamics (Fig 1A).

We applied the Galerkin finite element formulation in AngioMT using first order triangular elements to compute the solution to the stationary mass transport equation or Fick’s 2^nd^ law without any fluid convection (Eq 1).


$$0=-\nabla \cdot \left(-D_{O_{2}}\nabla C_{O_{2}}\right)+R_{O_{2}}$$ (1)

Galerkin finite element formulation is a common finite element strategy used for elliptical PDEs like Eq (1) and yields stable solutions over entire domains [19,20]. We solved for species concentration within the microvascular networks with the program assuming that bulk reactions do not occur in the endothelial cells and that the networks were permeable to the diffusing species. For the the connected vessel domain, Eq 1 modifies to the following equation along with the boundary conditions:

$$0=-\nabla \cdot \left(-D_{O_{2}}^{vessel}\nabla C_{O_{2}}^{vessel}\right)$$ (2)

$$C_{O_{2}}^{vessel}=C_{inlet}^{vessel}\forall \;\text{inlet}\;\text{nodes}$$ (3)

$$\left(-D_{O_{2}}\nabla C_{O_{2}}\right)\cdot \hat{n}=-P_{O_{2}}^{vessel}\forall \;\text{edge}\;\text{nodes}$$ (4)

Where, $D_{O_{2}}^{vessel}$ is the diffusion coefficient of oxygen within the vessels, $C_{O_{2}}^{vessel}$ is its concentration and $P_{O_{2}}^{vessel}$ is the permeability of species across the vascular wall (negative sign implies outward movement). A Dirichlet type boundary condition (2) was applied at nodes which were connected to the inlets, while a Neumann type boundary condition (3) was prescribed at the interface nodes of microvessel and tissue phases due to the permeability of vessel networks. Once we obtained the nodal concentration values within the microvessel phases, we then solved the steady state two-dimensional mass transport with reaction in the tissue phase:

$$0=-\nabla .\left(-D_{O_{2}}^{tisue}\nabla C_{O_{2}}^{tissue}\right)+R_{O_{2}}^{tissue}$$ (5)

$$C_{O_{2}}^{tissue}=C_{edge}^{vessel}\forall \;edge\;nodes$$ (6)

Where, $D_{O_{2}}^{tissue}$ is the diffusion coefficient of oxygen within the tissue, $C_{O_{2}}^{tissue}$ is its concentration and $R_{O_{2}}^{tissue}$ is the rate of removal/production of the species in the bulk tissue. Additionally, Dirichlet boundary condition was applied at edge nodes which were extracted from the previous step of obtaining concentration values at edge nodes of microvessel phases. Although we have used a Dirichlet type boundary condition for the tissue phase, AngioMT can be setup to use a Neumann type boundary condition as well. However use of Neumann boundary condition would require estimation of net oxygen consuption (flux) of the surruonding tissue for high oxygen consuming tissues. The reaction term $R_{i}^{tissue}$ can be of any order (ex. first order, Michaelis-Menten kinetics etc.); we applied the *Picard’s iteration scheme* to solve the non-linear differential equations that might arise from the complexity of the $R_{O_{2}}^{tissue}$ term.

Once nodal and element information was obtained from the meshing, we generated the element stiffness and force matrices in AngioMT according to the Galerking finite element formualtion of Eq 1. Elemental stiffness (*K*_*element*_) and force matrices (*F*_*element*_) were then mapped to the global stiffness (*K*_*global*_) and force matrices (*F*_*global*_) respectively. The final solution was calculated by performing the matrix inversion operation:

$$C_{O_{2}}={\left[K_{global}\right]}^{-1}\left[F_{global}\right]$$ (7)

After nodal concentration values were obtained at all nodes of the region of interest, the program calculates auxiliary variables like flux vectors, area-averaged mass transport etc (Fig 1A).

### AngioMT extracts domain information and assesses computational mesh quality

As described above, we applied the image labeling algorithm to the segmented images in AngioMT, extracted vascular domains (connected, disconnected and avascular tissue sections), and meshed them into distinct triangular mesh regions **(Fig 1D)**. Since the finite element method computes a solution on the nodes of any triangular element, the boundary conditions utilized in the transport dynamics were also prescribed as nodal conditions i.e., were applied on the nodes. Hence, after the mesh was structured, we also identified the nodes at the interfaces of the vessel and tissue domains for the purpose of applying boundary conditions **(Fig 1E)**. These nodes serve as edge nodes for applying a Neumann type oxygen permeabilty boundary condition (Eq 4) for the vessel domain. These nodes also acted as the Dirichlet type boundary condition for the tissue domain once oxygen values at the edge nodes were obtained from vessel domain (Eq 6). We also identified all nodes in the vessel domains at the left and right edges of the images, which were considered as oxygen inlets in our analysis. These nodes served as the inlet nodes where a Dirichlet type boundary condition for oxygen concentration were prescribed (Eq 3).

Next, we set out to test the quality of the mesh as low-quality elements may cause poor interpolation of the computed results as well as introduce numerical inaccuracies [21]. Triangular meshes are one of the most common element types used in numerical modeling schemes to discretize surfaces into elements [22]. To evaluate whether the mesh we generated using the *Im2mesh* routine in AngioMT was able to generate high quality triangular meshes, we calculated the *Element Quality* parameter for each element according to the following relation [23]:

$$\;Element\;Quality\;=\frac{4\sqrt{3}}{3}\frac{A}{||Edge_{RMS}|{|}^{2}}$$ (8)

Where, *A* is the area of the triangular element and ||*Edge*_*RMS*_|| is the root mean square edge length $\left(||Edge_{RMS}||=\sqrt{\frac{a^{2}+b^{2}+c^{2}}{3}}\right)$. Even though a perfect triangular element (equilateral triangle) is valued at unity, average element qualities of greater than 0.9 are acceptable threshold to obtain an accurate solution as this ensures removal of severely skewed elements (elements with obtuse angles) [23]. We calculated the element quality for the meshes generated using Delaunay Triangulation and found that the average quality scores were greater than 0.9 when a gradient limit of 0.2 was chosen for meshing **(Fig 1F)**. We also compared the element quality score distribution for multiple images and found that the meshing parameters were appropriate to generate meshes of quality with values close to unity **(Fig 1G and S1 Table)**.

AngioMT has been designed to process imaging data with minimal pre-processing making it easier to compute two phase oxygenations (tissue and vessel phases). The computed oxygen distributions are also numerically stable and were confirmed against a commercial transport solver **(S1 Fig)** [14].

### AngioMT predicts oxygen transport regulated by extent of vascularization

**Fig 2:**
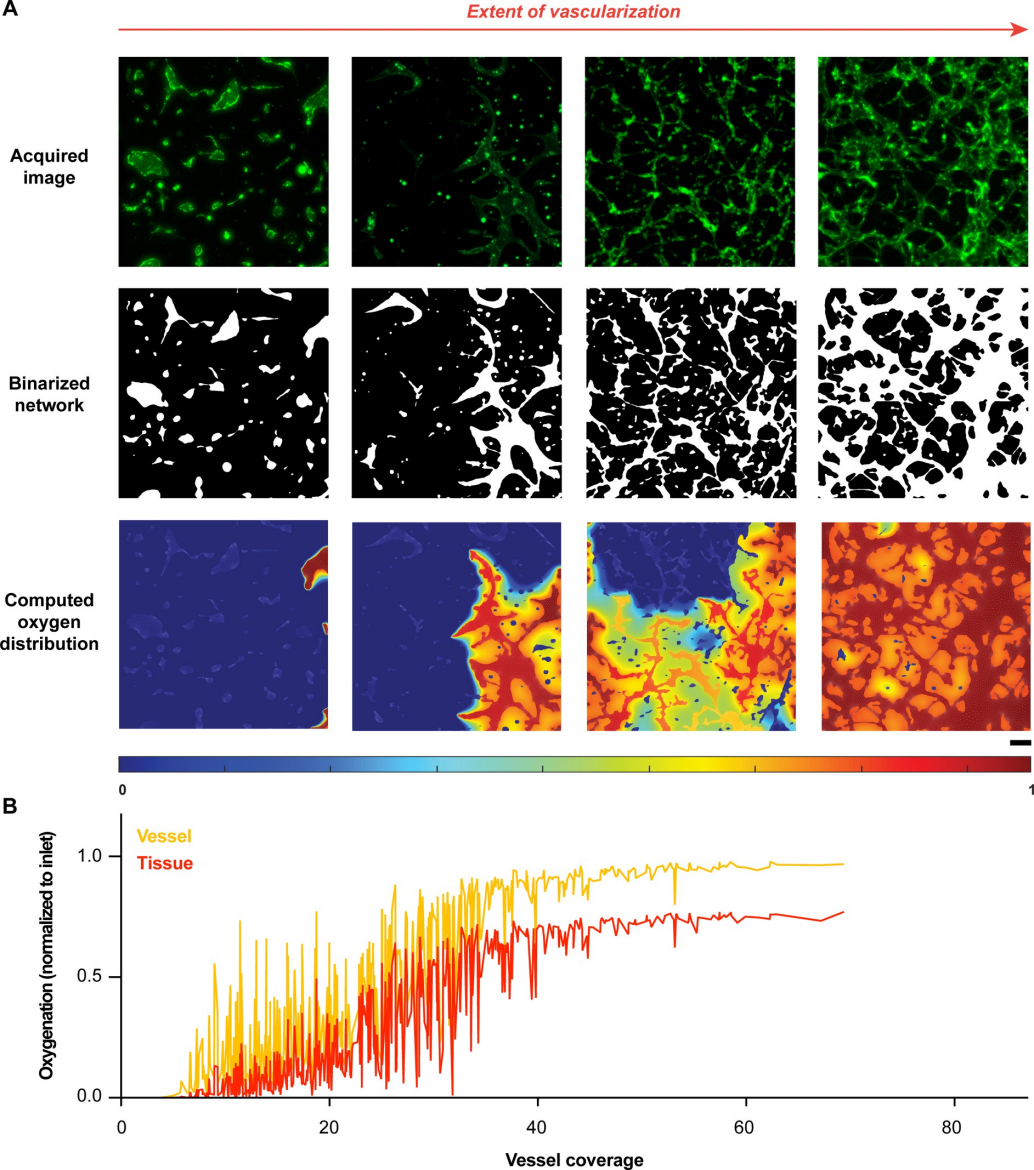
Assessment of oxygen transport through microvascular networks with a diversity of vascular coverage. **(A)** Oxygen distribution in microvascular networks with varied extents of vascularization (normalized to inlet oxygen concentration), described through fluorescence images, binarized images, and computed oxygen distribution (scale bar: 100 μm). **(B)** Vessel and tissue oxygenation computed by AngioMT for over 500 vascular networks formed with different experimental conditions, plotted against the corresponding vessel coverage values.

Since AngioMT could solve oxygen transport in complex vascular network domains, we were now inspired to apply AngioMT to describe the spatial distribution of oxygen over a vast range of vascular networks. Various factors, like stromal cell co-culture, type of endothelial cells, matrix stiffness, growth factors etc. affect the spatial distribution and functioning of microvascular networks [10,24,25]. Therefore, we analyzed over 500 images of vascular networks with different experimental conditions that have been previously published by our group [11,26] and examined if AngioMT was sensitive to the extent and morphological variability of the vascular networks **(Fig 2A and 2B)**. For example, for conditions that resulted in extremely low vascularization with disconnected networks, AngioMT predicted a poor oxygen delivery to the tissue (Fig 2A; first column). Similarly for experimental conditions that resulted in some vascularization, AngioMT estimated a higher oxygen concentration in the tissue domain (Fig 2A; second column). Finally, moderately, and densely vascularized conditions also exhibited a proportional increase in the average tissue oxygenation (Fig 2A, third and fourth columns).

Overall, both vessel and tissue oxygenation followed a near sigmoidal trend when computed against the vessel coverage of all the 500 images with varying extents of vascularization **(Fig 2B)**. We also observed that when vessel coverage was below 40%, the oxygen transport was fluctuating **(Fig 2B)**. This may be expected as vessel coverage values are representative of the total vessel area in an image, however, they are not representative of how these vessels are spatially-distributed within an image. It is possible that even though there were conditions with moderate vascularization, the vessels were distributed such that they received lower oxygen from the inlets (Fig 2A, third column). Interestingly, at higher values of vessel coverage, the vessel and tissue oxygenation reached saturation, suggesting that vessel coverage values greater than 40% resulted in well-distributed networks within each image and received sufficient oxygen from the inlets. Overall, our program revealed high sensitivity of computed oxygenation to a morphological marker often used in scientific literature [11,18] to evaluate the performance of a vascular network (**S2 Table**).

### AngioMT predicts oxygen distribution comparable to experimentally measured within vasculature *in vivo*

**Fig 3:**
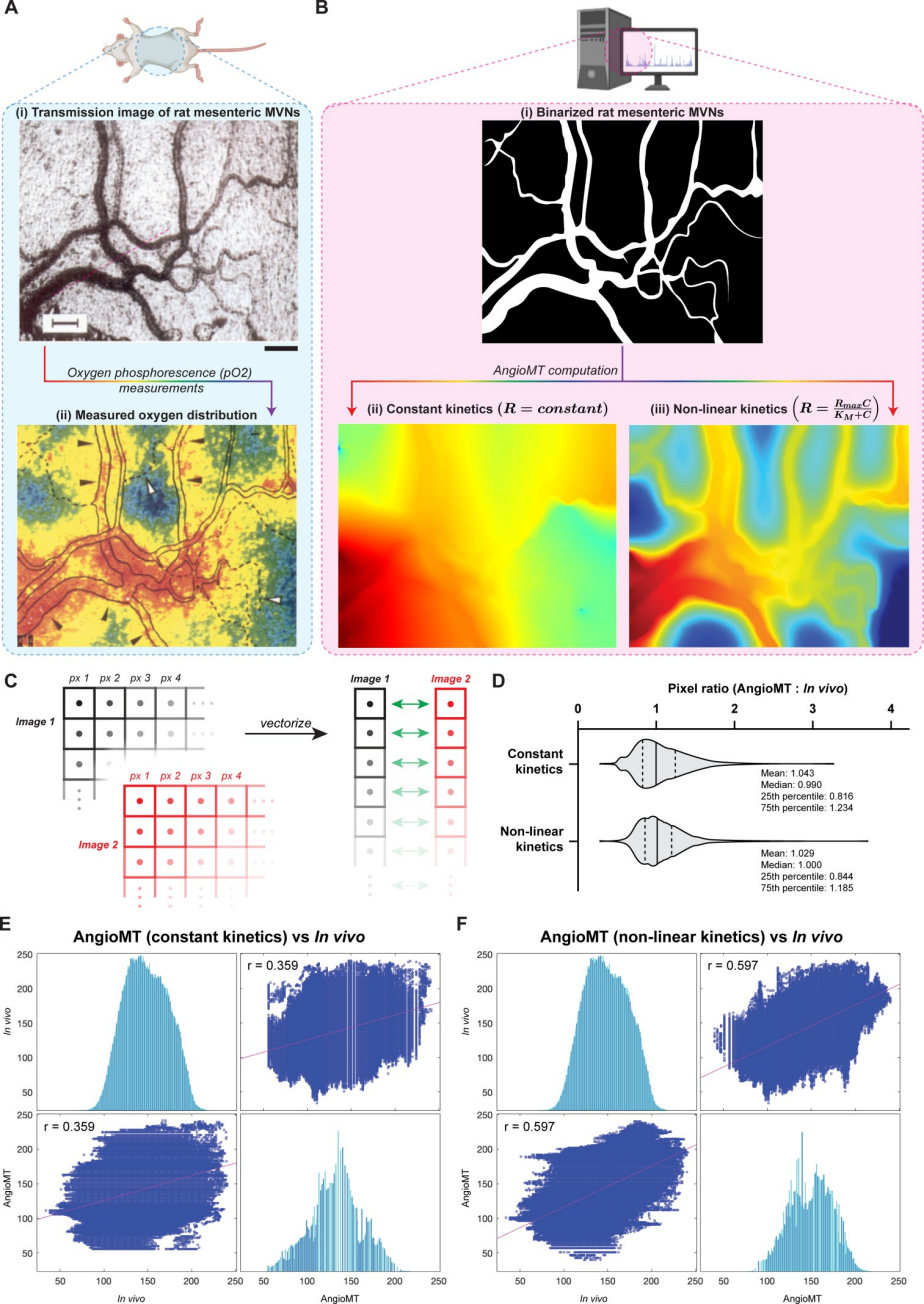
Evaluation of predicted oxygen distribution in rat mesentery against *in vivo* experimental measurements. **(A)** *In vivo* imaging of oxygenation around rat mesenteric microvascular networks; (i) Brightfield image of rat mesenteric microvascular networks taken from Itoh et al [27] (scale bar: 100 μm), (ii) Experimentally obtained color micrograph of oxygen distribution within the rate mesenteric microvasculature reported by Itoh et al [27]. **(B)** Computational prediction of *in vivo* results; (i) Binarized image of the brightfield rat mesenteric microvascular networks shown in A-(i) for computational analysis with AngioMT, vessel and tissue oxygenation as predicted by AngioMT assuming (ii) constant reaction kinetics, and (iii) enzymatic/non-linear kinetics in the mesenteric tissue. **(C)** Schematic of image processing methodology for comparison of predicted and experimental results. Colored images are resized to same pixel sizes and converted 8-bit grayscale images followed by vectorization of pixel values and subsequent statistical analysis. **(D)** Pixel-to-pixel ratios between AngioMT and *in vivo* results for constant and non-linear kinetics. Pearson correlation plots between predicted oxygen distributions by AngioMT and *in vivo* images assuming **(E)** constant (r = 0.359) and **(F)** non-linear kinetics (r = 0.597).

After evaluating the sensitivity of AngioMT to the extent of vascularization, we were then motivated to validate the oxygen distribution values predicted by AngioMT against experimentally-measured values in microvascular networks found *in vivo*. To compare the oxygen distributions between computational and experimental approaches, we acquired brightfield imaging data from rat mesenteric microvasculature, provided in literature [27,28] **(Fig 3A-i)**. The experimentally observed oxygen distribution values suggested that blood vessels were the oxygen source and the transport of oxygen was into the tissue as regions within and around the vessels were highly saturated with oxygen **(**red/yellow zones, **Fig 3A-ii)**. We applied the imaging data into our AngioMT software, which successfully generated a binarized image of the microvasculature **(Fig 3B-i).** To compare *in vivo* results and computed AngioMT results pixel-to-pixel **(Fig 3C)**, we first resized images to the same pixel size for all conditions and then converted them to 8-bit grayscale images in ImageJ. To normalize the differential imaging and measurement modalities, we matched the image histograms of AngioMT results in reference to the *in vivo* oxygen distribution using the MATLAB *imhistmatch* function [29]. These scaled, histogram matched images were then converted to column vectors of all pixel values which allowed us to generate pixel-wise ratios between AngioMT results and *in vivo* observations **(Fig 3D and S3 Table)**. When we computed the oxygen distribution in the vessel and tissue domains assuming constant reaction kinetics in tissue **(Fig 3B-ii)**, the distribution of pixel-to-pixel ratios was centered around unity suggesting that the averaged computational predictions overlapped the *in vivo* images. However, the Pearson cross-correlation was found to be modest (r = 0.359) possibly because of the simplicity of our computational model. Since oxygen consumption in tissues is known to follow Michaelis-Menten reaction kinetics [30], we now also integrated the possibility to compute more complex, non-linear enzymatic reaction kinetics (Michaelis-Menten reactions) in our AngioMT software, assuming that:

$$R_{O_{2}}=\frac{R_{max}C_{O_{2}}}{K_{M}+C_{O_{2}}}$$ (9)

Interestingly, when we applied this non-linear kinetics, we again found that the distribution of pixel ratios to be closer to unity, with a reduced variance relative to constant reaction term (Fig 3D). Interestingly, the cross-correlation of computed oxygen distribution with *in vivo* data improved nearly two-fold when a non-linear reaction term was included relative to it being a constant **(Fig 3E and 3F)**. Therefore, even though our computational model solves the transport physics using a 2D assumption and does not include some possible contribution of convective flow [31], it may still be applied in MPS systems to evaluate vascular networks with an additional possibility to include these phenomena to enhance this correlation further.

### AngioMT calculates ancillary variables of mass transport

**Fig 4:**
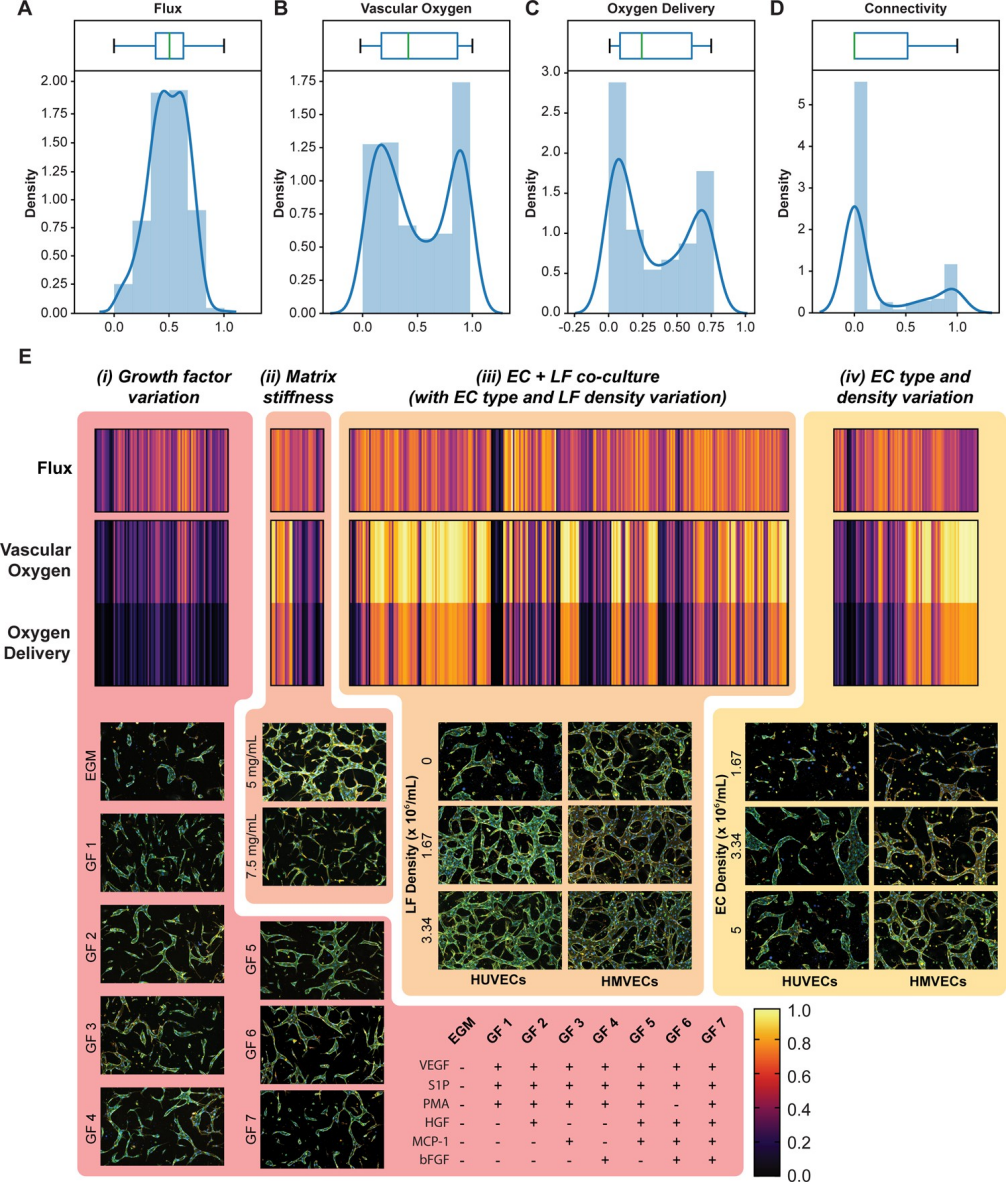
AngioMT calculates ancillary variables of oxygen transport. Box and density plots of **(A)** ‘Flux’, **(B)** ‘Vascular Oxygen, **(C)** ‘Oxygen Delivery’ and **(D)** ‘Connectivity’. **(E)** Heatmap showing distributed values of ancillary variables calculated by AngioMT for a 500 image dataset encompassing different microvascular networks produced by varying (i) growth factor; (ii) fibrin matrix stiffness; (iii) endothelial cell and lung fibroblast co-cultures; and (iv) endothelial cell type and density.

In the biological assessment of vascularized tissue microenvironments (such as tumors, islets, liver etc), while bulk oxygen distribution may provide a first-order metric of tissue health, granular information, such as, vascular oxygen potential and oxygen delivered to the tissue, may expand our knowledge of how a vascular network specifically regulates the microenvironment. Therefore, we next computed several ancillary transport metrics with AngioMT that are representative of vascular network connectivity and mass transport in the context of microvascular networks, but typical commercial solvers may not provide. For example, we could easily compute total oxygen flux that represents the rate of oxygen delivery of a vascular network (**Fig 4A**). Correspondingly, we were also able to generate ‘arrow’ plots (**S2 Fig**) or ‘contour’ plots (**S3 Fig**) for a visual depiction of flux or oxygen transport, which revealed the directionality of oxygen transport or its spatial gradients, respectively.

We next set out to examine the oxygen content specifically within the vascular domain of the network, and oxygen delivered specifically to the solid tissue domain, as these two measures are physiologically-relevant and may predict the health of a vascularized tissue. We first calculated “Vascular Oxygen” as the area averaged oxygen concentration (normalized to inlet oxygen concentration) within the vessel domain and is indicative of the network’s capacity to carry oxygen (**Fig 4B**). Unlike flux, the distribution of this parameter was bimodal as our image datasets had more instances of slightly vascularized and highly vascularized samples. This was also seen in Fig 4B, where the vessel oxygenation values were saturating at low and high values of vessel coverage.

We then also computed ‘Oxygen Delivery’, which is the area averaged oxygen concentration in the tissue/hydrogel domains (normalized to inlet oxygen concentration, **Fig 4C**). This metric represents the spatial distribution of oxygen within the tissue region and indicates the net oxygen delivered to the tissue through the networks. As before, the distribution of this parameter was bimodal and followed the same reasoning as ‘Vessel Oxygen’.

Since the main purpose of microvascular networks is to deliver oxygen to the surrounding tissues, calculating these two metrics may provide information regarding the extent of oxygen delivery, that may be required to evaluate the performance and biological function of vascular networks.

Finally, we also computed network connectivity (line averaged oxygen concentration normalized to inlet) in the vessel representative of the directional end-to-end transport of oxygen when only one inlet is assumed (left edge). This measurement correlates to applied experimental techniques like dextran perfusion capacity, or connectivity measurements reported in experimental literature [32]. Interestingly, even though our analyses of this measure on the 500 samples resulted in a bimodal distribution, the distribution was extremely skewed towards the lower end suggesting our networks are mostly poorly connected (**Fig 4D**). Although well connected networks are desired, networks with lower connectivity are still able to deliver oxygen if they are situated close the inlets (as demonstrated by ‘Vessel Oxygen’ and ‘Oxygen Delivery’ parameters). Additionally, well connected networks might not necessarily mean well distributed networks, further suggesting that this metric might not be representative of transport characteristics of the network, which is normally distributed in our samples.

Since vascular networks may vary depending on the amount of growth factors, matrix stiffness, presence or absence of fibroblasts, cell density and type etc., and it is of wide interest amongst scientists to perform parametric investigations to characterize the influence of these variables on vascular network’s capabilities to oxygenate a tissue microenvironment [10], we also assessed these metrics when we arranged the vascular network samples based on variation of these parameters (**Fig 4E and S4 Table**). Interestingly, we found that varying physiological parameters like growth factor concentrations (Fig 4E-i), matrix stiffness (Fig 4E-ii), stromal cell density (Fig 4E-iii), and endothelial cell type (Fig 4E-iv) resulted in different extents of vessel and tissue oxygenation. Interestingly, within each experimental condition, the Vessel Oxygen and Oxygen Delivery values were varying between images, further demonstrating the spatial-recognition of AngioMT in differentiating images within each experimental condition. Taken together, when used combinatorically, these metrics may provide a quantitative and more detailed assessment of delivery of cells, molecules, drugs, and toxins in a vascularized tissue in health and disease.

### Evaluating organ-scale oxygenation of multicellular tissues using AngioMT

**Fig 5:**
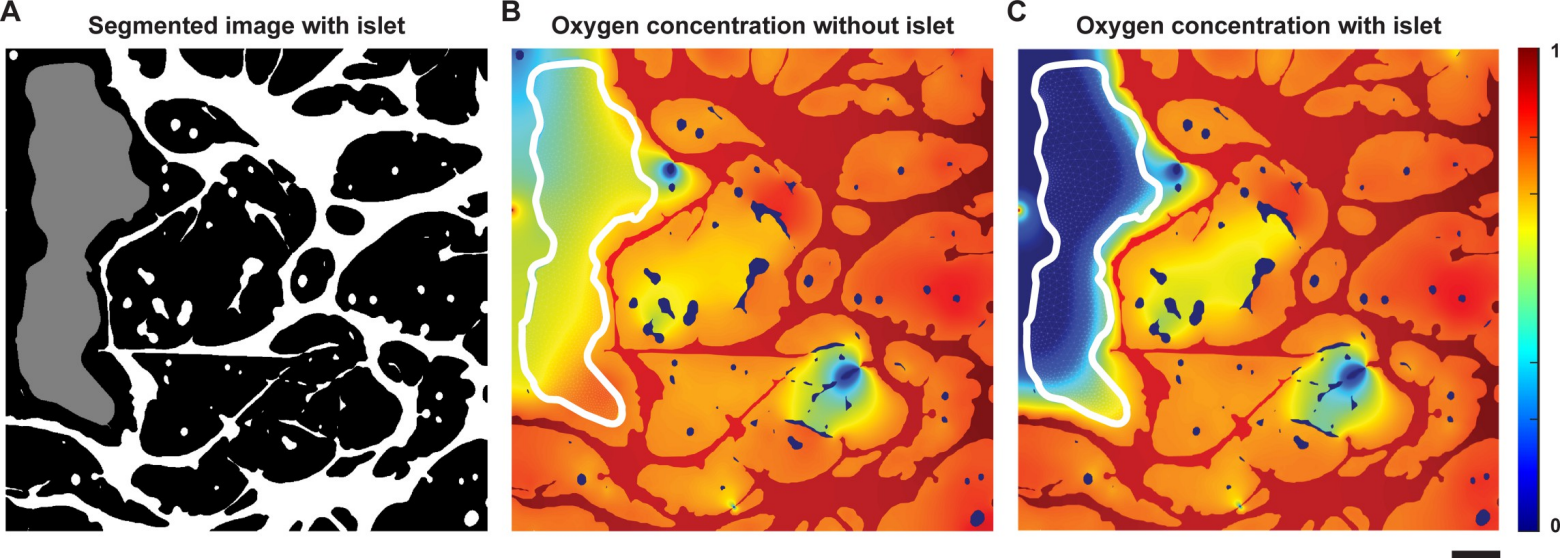
Oxygen transport in the microvascular niche of a multicellular islet (organ). **(A)** Binary image showing the microvascular network (white) with an artificially generated islet (gray). **(B)** Oxygen distribution in the tissue and islet domains assuming islets are part of the tissue (no metabolic differences in oxygen consumption). **(C)** Oxygen distribution in the tissue and islet regions assuming a more metabolically active islet (scale bar: 100 μm).

Since AngioMT package has been built to isolate the solid tissue domain from the vessels in the network, we finally set out to demonstrate the ability of our program to predict oxygen transport with and without the presence of the solid tissue consisting of any co-cultured cell types. As a representative case study, we assumed an islet as a solid tissue in one of the images from our dataset and computed the oxygen distribution patterns (**Fig 5A**). Since the metabolic and kinetic activity of an islet and the surrounding tissue are different, we extended the software to define the multiple kinetic rate parameters (reaction rate constants) corresponding to each tissue/ cell type in an image.

When the region denoted by the islet was considered part of the tissue domain (and not a metabolically different tissue), AngioMT predicted higher tissue oxygenation values as expected as the metabolically active cell type was absent (**Fig 5B**). Contrastingly, when the islet was assumed to be more metabolically active than the surrounding tissue, AngioMT predicted lower tissue oxygenation levels which is expected (**Fig 5C**), showing that the software is able to predict the changes in oxygen transport phenomena of a vascular network due to additional cell or organ-types present in a tissue microenvironment. Although we have used vascularized islets as an example case study, this methodology can be extended to other vascularized organ tissues like brain, liver, lungs etc. Predictions made using AngioMT can further be made more physiologically relevant by studying oxygen transport through more complex, multi-tissue systems that might arise in cancerous conditions like ovarian cancer and surrounding ovaries. Consequently, the model may provide a tool to evaluate models of cell transplants, other regenerative medicinal products, and vascularized organoids and muti-organ-chips.

## Conclusion

In this study we demonstrate AngioMT, a spatially-informed computational mass transport solver to evaluate tissue and vascular oxygentaion levels in microcirculation. Using routinely acquired 2D confocal images, AngioMT demonstrates and analyzes mass transport within a heterogenous system of microvessels. The image-to-physics methodology allows us to incorporate the spatial complexity that ultimately dictates the spatiotemporal movement of dissolved nutrients and factors. The feature extraction principles to detect edges and edge nodes enable effective application of boundary conditions in an automated manner. AngioMT can also incorporate complex, non-linear reactions kinetics like Michaelis-Menten enzyme kinetics to predict a more physiologically relevant oxygen distribution using iteration schemes to compute final concentration values. Finally, AngioMT also calculates ancillary variables like flux vectors, tissue oxygenation, vascular oxygen potential etc. which can provide additional information for assessing therapeutic interventions in the microvascular niche. It is important to note that in our analysis, we have ignored the diffusion barrier present due to the endothelial cell thickness since our computational domains were of the order of 1000 μm while endothelial thickness was ∼5–10 μm. However, AngioMT is capable of including the added diffusion barrier due to the endothelium when needed. Although AngioMT uses 2D images currently, it could still be used as a physiologically relevant first order approximation of oxygen transport in vascularized systems to understand their biological function. Contemporary imaging techniques, for example, Photoacoustic Lifetime Imaging (PALT), Positron Emission Tomography (PET), Nuclear Magnetic Resonance (NMR) etc., can measure oxygen tension in a tissue in 3D with higher depth resolution, relative to a more classical technique that we have utilized for validation of computed oxygen profiles in AngioMT. However, it is important to note that the more recent approaches to visualize oxygen transport are mostly restricted to a local region, and literature is very sparse which shows oxygen distribution maps over large tissue regions that can be successfully used to validate the model with higher rigor. Additionally, since 2D imaging is easy and faster to perform both *in vitro* and *in vivo*, and 2D data is still a gold standard in a variety of clinical decision-making, we have strategically created AngioMT as a 2D image-to-physics solver first. However, with current computational hardware and available speeds, AngioMT can be extended to 3D systems as well for modeling more complex, three dimensional imaging datasets, when needed.

AngioMT is a computational tool that is expected to be used as a performance evaluation strategy for microvascular networks in microphysiological systems. The spatial assessment of oxygen tranposrt offered by AngioMT bolster its application as a tool for “scoring” networks. AngioMT was recently used by us in conjunction with other physiological vessel metrics, for example vessel length, area, segment diameter, number of branchpoints, to create more physiologically relevant scoring metrics [11]. We have recently also demonstrated another application where data from AngioMT served as the ground truth data to create an anrtificial-intelligence based program to evaluate vascular networks in biological systems [26].

Ultimately, this tool will enable vascular scientists and engineers to visualize nutrient or drug transport through complex, heterogenous microvascular networks. Furthermore, unlike commercailly available packages, the AngioMT procedure can be automated to analyze large amount of data. Automatation of this computational methodology is possible with minimal interventions for screening high throughput data, which can eventually be incorporated with next generation machine learning techniques. This analytical approach has potential to enable clinicians and scientists to assess whether the extent of vascularization is enough for delivering and retreiving important factors like oxygen, nitric oxide, growth factors, and drugs, which can ultimately affect the success of clinical procedures like organ transplants and embedded biosensors.

## Supporting information

S1 Fig Advantages of AngioMT over COMSOL. Normalized **v**essel oxygenation computed using **(A)** COMSOL and **(B)** AngioMT. Unlike COMSOL, AngioMT can incorporate contributions from disconnected vessels on vessel oxygenation and simulates tissue oxygenation as well (scale bar: 100 μm).
(TIF)

S2 Fig AngioMT can generate ‘arrow’ plots for understanding the directionality of oxygen transport. In addition to species concentrations, AngioMT can also provide elemental flux values as well as flux vectors (shown in insets i, ii and iii; scale bar: 100 μm; inset scale bar: 10 μm inset).
(TIF)

S3 Fig AngioMT can plot contours for alternate visualization of tissue oxygenation. In addition to flux vectors, AngioMT can also produce contour plots of oxygen concentrations within the tissue domain (scale bar: 100 μm).
(TIF)

S1 Table Element quality analysis of computational mesh created on three representative images of vascular networks. (XLSX)

S2 Table Image-wise vessel and tissue oxygenation computed by AngioMT for vascular networks formed with different experimental conditions. (XLSX)

S3 Table Grayscale pixel values extracted from representative in vivo image as well as corresponding AngioMT results with constant and non-linear kinetics. (XLSX)

S4 Table Image-wise ancillary variables (Vessel oxygen, Oxygen delivery, Flux and Connectivity) computed for vascular networks using AngioMT. (XLSX)
