# Supplementary figures and images for: AngioMT: A MATLAB based 2D image-to-physics tool to predict oxygen transport in vascularized microphysiological systems
Source: PLoS One. 2024 May 15;19(5):e0299160. doi: 10.1371/journal.pone.0299160 (PMC11095698; doi:10.1371/journal.pone.0299160)

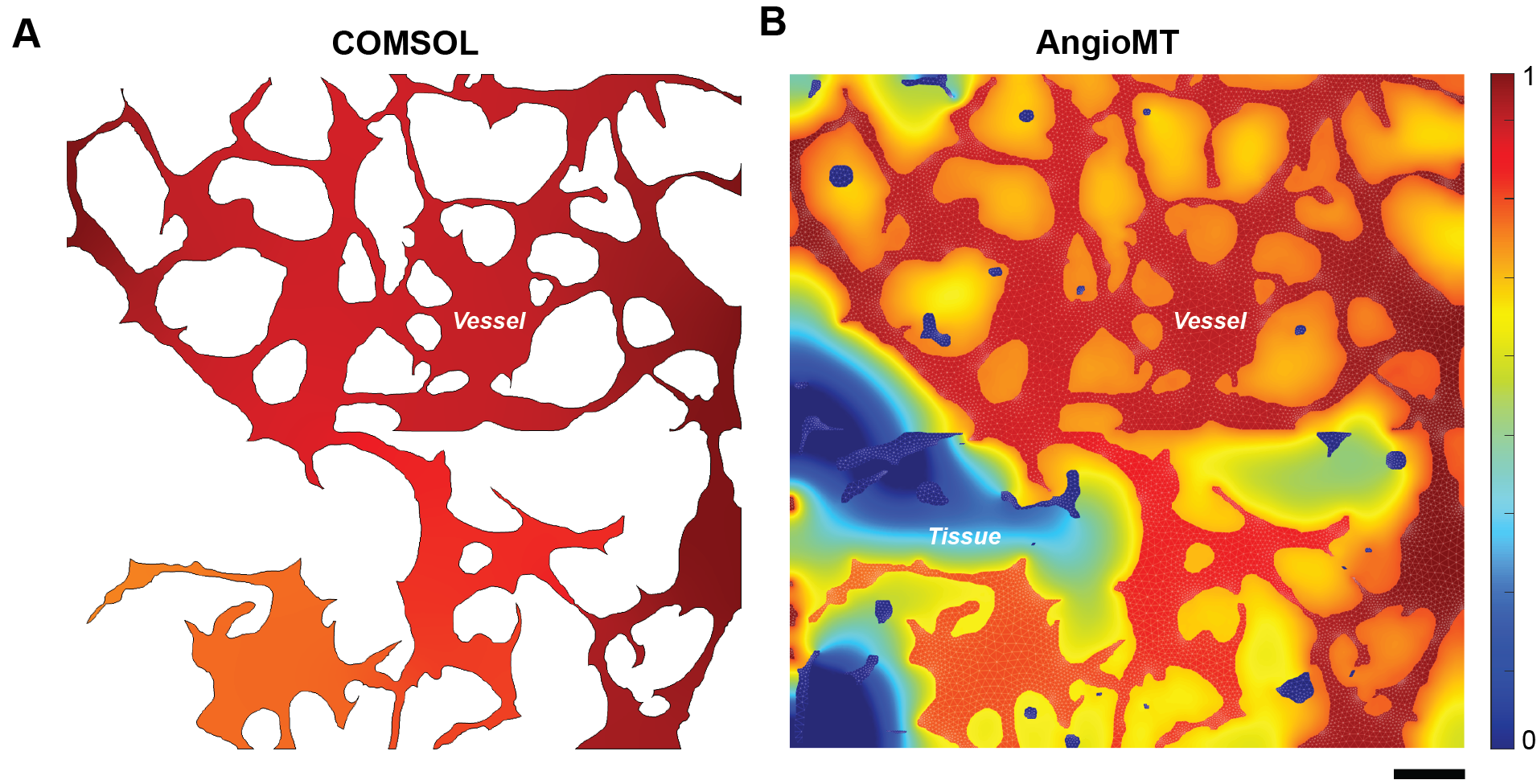

Supplement: S1 Fig — Normalized vessel oxygenation computed using (A) COMSOL and (B) AngioMT. Unlike COMSOL, AngioMT can incorporate contributions from disconnected vessels on vessel oxygenation and simulates tissue oxygenation as well (scale bar: 100 μm). (TIF) [file pone.0299160.s001.tif]

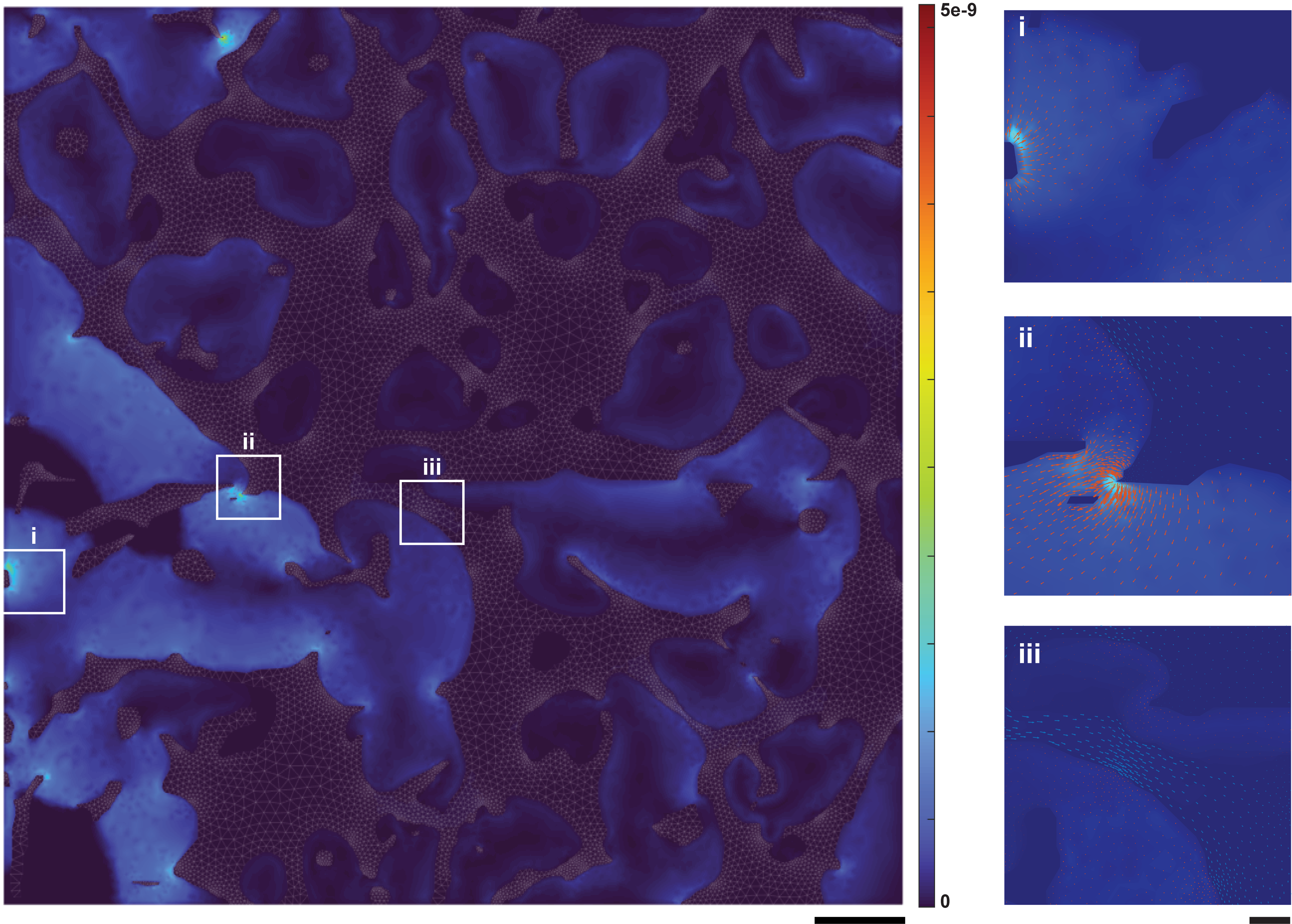

Supplement: S2 Fig — In addition to species concentrations, AngioMT can also provide elemental flux values as well as flux vectors (shown in insets i, ii and iii; scale bar: 100 μm; inset scale bar: 10 μm inset). (TIF) [file pone.0299160.s002.tif]

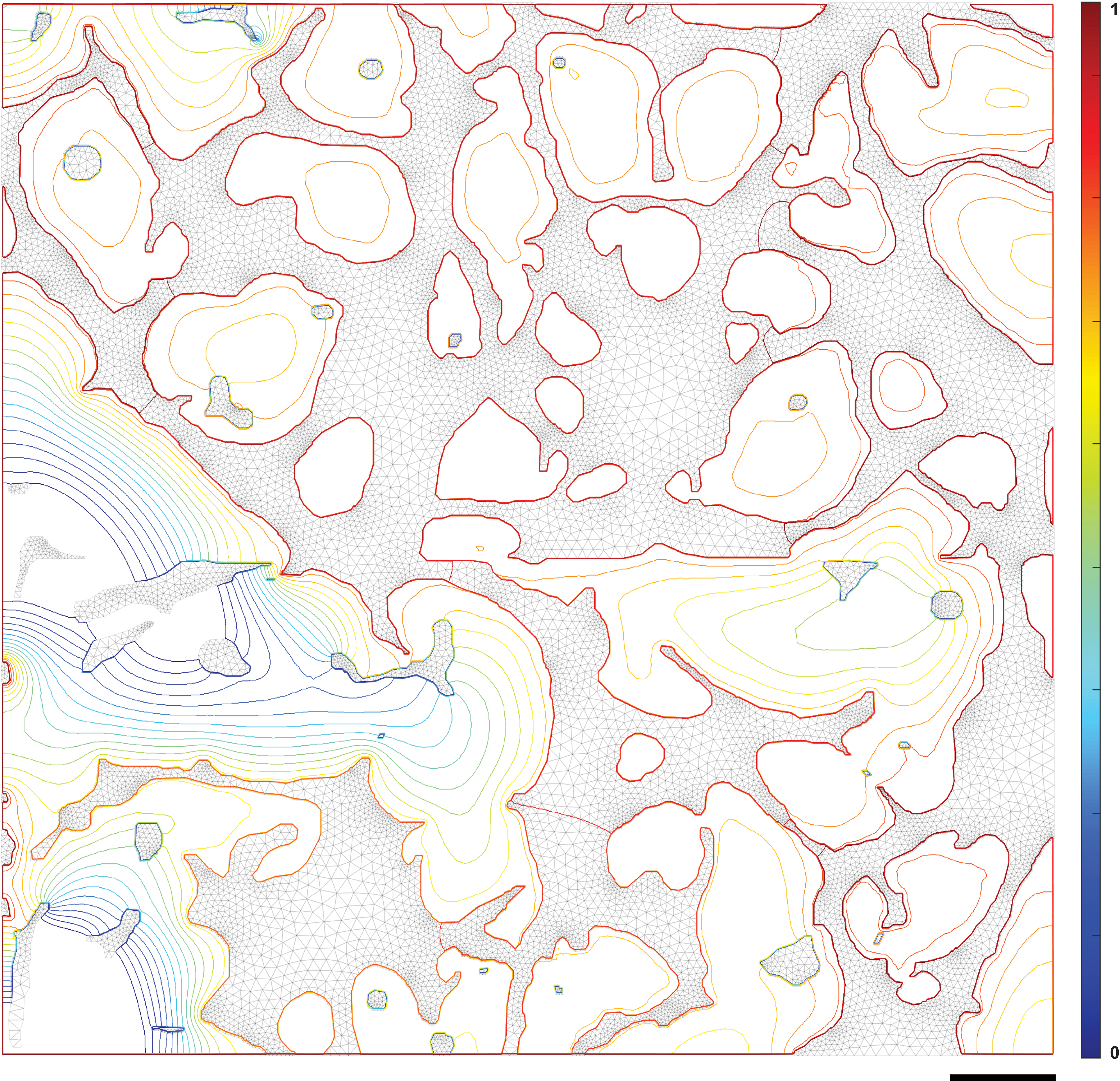

Supplement: S3 Fig — In addition to flux vectors, AngioMT can also produce contour plots of oxygen concentrations within the tissue domain (scale bar: 100 μm). (TIF) [file pone.0299160.s003.tif]
